# Psychiatric comorbidities of chronic migraine in community and tertiary care clinic samples

**DOI:** 10.1007/s10194-012-0480-3

**Published:** 2012-09-01

**Authors:** Antonio Lucio Teixeira, Esther Angélica Coelho Costa, Ariovaldo Alberto da Silva, Igor Alvarenga Moreira dos Santos, Rodrigo Santiago Gómez, Arthur Kummer, Edward C. Lauterbach

**Affiliations:** 1 Headache Clinic, Neurology Unit, University Hospital, Universidade Federal de Minas Gerais (UFMG), Av. Alfredo Balena 110, Belo Horizonte, MG 30130-100 Brazil; 2 Neuropsychiatry Branch, Neurology Unit, University Hospital, Universidade Federal de Minas Gerais (UFMG), Av. Alfredo Balena 110, Belo Horizonte, MG 30130-100 Brazil; 3 Department of Psychiatry and Behavioral Sciences, Neurology Section, Mercer University School of Medicine, 1400 Coleman Avenue, Macon, GA 31201 USA; 4 Department of Internal Medicine, Neurology Section, Mercer University School of Medicine, 1400 Coleman Avenue, Macon, GA 31201 USA; 5 Serviço de Neurologia, Hospital das Clínicas, Universidade Federal de Minas Gerais, Av. Alfredo Balena, 110, Belo Horizonte, MG 30130-100 Brazil

**Keywords:** Migraine, Chronic migraine, Comorbidity, Depression, Anxiety

## Abstract

Although the association between episodic migraine and psychiatric comorbidities is well documented, few studies have focused on the comorbidity with chronic migraine (CM) and discrepancies exist between population-based and clinic-based data. The objective of this study is to compare demographic and psychiatric comorbidity correlates between CM samples drawn from the community and tertiary care. All inhabitants from a city borough were interviewed for the presence of headaches occurring 15 or more days per month. CM was diagnosed after subjects had been interviewed and examined by a headache doctor. Participants were also assessed with a structured interview by a psychiatrist, who assigned diagnoses based on the DSM-IV. The same investigators assessed all patients consecutively seen in a university-based outpatient headache center over a 4-month period. The samples consist of 41 individuals from the community and 43 from the headache center. Sociodemographic profiles were similar between groups with the exception of the mean number of years of formal education. Among individuals from the community, psychiatric diagnoses were present in 65.9 % of cases, relative to 83.7 % in those from the headache center (*p* = 0.06). Phobias (41.9 vs. 29.3 %) and depression (32.6 vs. 29.3 %) were more frequent in patients from the headache center, but this difference did not reach statistical significance. Thus the frequency of psychiatric disorders in patients with CM was elevated in both settings, being higher in the specialty care clinic.

## Introduction

Migraine is the leading neurological cause for seeking medical care [1], and is associated with significant disability in the sufferer [2]. The greatest impact is on migraineurs with headaches on more days than not [3], a condition defined as chronic migraine (CM) [4].

CM is defined as at least 15 days of headache per month in which at least eight of the days fulfill migraine criteria and/or are treated with specific migraine medications, in the absence of a diagnosis of medication overuse headache [4]. Patients with CM often had a history of episodic migraine that began in adolescence or early adulthood, reporting a process of transformation marked by headaches that become more frequent over years [5]. Among migraineurs, defining risk factors for CM, or for the progression of episodic migraine to CM, is an issue of scientific and public health interest [6]. Identified risk factors include medication overuse, obesity, sleep problems, and psychiatric comorbidity [7–12].

Studies in both community and tertiary settings have demonstrated an association between migraine and several psychiatric conditions [8, 9]. However, the frequency of psychiatric disorders in both setting has not been compared before in a single study. Furthermore, differences in methods of studies based in community or tertiary centers prevent appropriate comparison. Population studies fail to conduct face-to-face assessments, while clinic-based studies carry the potential for selection bias.

Studies focusing on best methods to address this gap are of interest, and one strategy is to compare data obtained from the community with those from specialty care, where methods of collection have been virtually identical, and that was the scope of this study. We compared demographic data and psychiatric comorbidity in a sample of individuals with CM from the community with another from a tertiary care clinic. In light of the fact that patients suffering from migraine and comorbid psychiatric disorders are greater health-care service users [8], we hypothesized that the frequency of psychiatric disorders, notably depression, is higher in patients followed in tertiary care.

## Methods

### Population study

Community data were gathered in Capela Nova, a city from the state of Minas Gerais, Brazil. According to the 2000 Brazilian census, its population was 2,066 inhabitants (1,631 over the age of 10 years). The present study is part of an observational, cross-sectional and population-based study conducted in two phases [14, 15].

Initially, community health workers from the Family Health Program directly interviewed all inhabitants aged 10 years or older for headache symptoms in the previous year. The Family Health Program works through family health-care teams which are composed of one physician, one nurse, one auxiliary nurse, and four to six community health workers, and are assigned to specific geographical areas with defined populations of 600–1,000 families. Activities provided by family health-care teams take place at primary care facilities, in patients’ homes, and in the community [16].

In the first phase of our study (screening phase), trained community health workers screened for the occurrence of headaches using the following question: “Have you had any headache episode over the last 12 months?” Those who screened positive were asked about headache frequency in the past month, and those reporting 15 days or more of headache were offered in-person assessment by neurologists with expertise in headache medicine. Three neurologists independently examined participants, and headaches were diagnosed according to the second edition of the International Classification of Headache Disorders. Assessments were conducted from December 2005 to March 2006. Detailed methods have been previously described [14, 15].

Subsequently, all individuals aged 18 years or older with CM were assessed for psychiatric comorbidities. Psychiatric assessment was performed by an experienced psychiatrist using the Mini International Neuropsychiatric Interview (MINI) [17]. Current psychiatric diagnosis was based on the DSM-IV-TR criteria [18].

### Clinic study

The same investigators involved in the community-based assessment used the very same procedures to diagnose consecutive patients attended at a university-based headache center in the first half of 2006. This center is located in Belo Horizonte and is the only headache clinic in the state of Minas Gerais, Brazil. Once identified, CM patients were assessed for psychiatric comorbidities as described above. All patients were older than 18 years.

The study followed the guidance of the regulatory norms of the Brazilian National Health Council (Resolution 196/1996) which is in accordance with the Helsinki Declaration. The protocol and all forms were reviewed and approved by the local ethics research committee.

### Statistical analysis

Demographics, clinical characteristics and comorbidities were compared between groups. Data were transferred to Epi-info 2000 by a study coordinator and analyzed using SPSS 12.0.

The relative frequencies of psychiatric comorbidities were stratified by headache type, and confidence intervals were calculated. The significance level was established at the 5 % level. Discrete data were compared between groups using the Chi-square test or the Fisher test (when anticipated values were small). For continuous non-parametric variables, the Mann–Whitney test was used.

## Results

In the community of 1,605 interviewed inhabitants, 57 (3.6 %) had headaches on at least 15 days for at least three consecutive months. These data have been published elsewhere [14, 15]. Of them, 43 (75.4 %) had CM and 41 consented to being assessed by the psychiatrist (95.3 % participation rate). In the headache center, 43 patients had CM (*N* = 453, 9.5 %) and all consented in participating in the psychiatric assessment.

Sociodemographic profiles were similar between groups with the exception of the mean number of years of formal education, lower in the community relative to the headache center (Table 1).

**Table 1:** Demographic characteristics in individuals with chronic migraine from the community and a clinic-based sample

	Community (*n* = 41)	Headache center (*n* = 43)	*p* value
Gender			
Men	7 (17.1 %)	2 (4.7 %)	0.09**
Women	34 (82.9 %)	41 (95.3 %)	
Education (years of study)			
≤8	26 (78.8 %)	5 (11.6 %)	**<**0.001*
9–11	5 (15.2 %)	15 (34.9 %)	
≥12	2 (6.1 %)	23 (53.5 %)	
Age			
Mean (SD)	41.2 (17.2)	35.7 (12.6)	0.19***
Range	13–73	18–63	

* Chi-square, ** Fisher, *** Mann–Whitney

Among individuals from the community, 65.9 % of cases were diagnosed with any current psychiatric disorder, relative to 83.7 % in those from the headache center (*p* = 0.06). The relative frequencies of some specific diagnoses were remarkably high in both groups, despite not being statistically different. In the headache center, the most prevalent disorders were simple phobia (41.9 %), generalized anxiety disorder (34.9 %) and major depression (32.6 %). In the community, the same disorders were also the most common ones: generalized anxiety disorder (39.0 %), phobias (29.3 %), and major depression (29.3 %). Bipolar disorder was not seen in the community and was diagnosed in two cases from the headache center. The frequency of antidepressants use was similar in the headache center (51.2 %) and in the community (44.4 %) (*p* = 0.51). Table 2 summarizes these data.

**Table 2:** Current psychiatric comorbidities in individuals with chronic migraine from the community and a clinic-based sample

	Headache center (*n* = 43)	Community (*n* = 41)	*p* value
Any diagnosis	36 (83.7 %)	27 (65.9 %)	0.06*
One or two psychiatric diagnoses	21 (48.8 %)	16 (39.0 %)	0.36
Three or more psychiatric diagnoses	15 (34.9 %)	11 (26.9 %)	0.42
Major depression	14 (32.6 %)	12 (29.3 %)	0.74*
Dysthymia	9 (20.9 %)	9 (22.0 %)	0.99**
Bipolar disorder	2 (4.7 %)	0 (0 %)	0.23**
Generalized anxiety disorder	15 (34.9 %)	16 (39.0 %)	0.69*
Specific phobia	18 (41.9 %)	12 (29.3 %)	0.23*
Obsessive compulsive disorder	9 (20.9 %)	10 (24.4 %)	0.70*
Somatization	7 (16.3 %)	3 (7.3 %)	0.31**
Eating disorders	2 (4.7 %)	1 (2.5 %)	0.99*
Alcohol abuse	0 (0.0 %)	2 (4.9 %)	0.23**

* Chi-square, ** Fisher

## Discussion

To the best of our knowledge this is the first study to compare the frequency of psychiatric comorbidity of CM in community and tertiary care clinic samples. The frequency of psychiatric disorders in CM was elevated in both settings, tending to be higher in the tertiary care sample.

While psychiatric comorbidity in episodic migraine has been well established in the literature [19], psychiatric disorders have been less studied in CM. Only a few studies have addressed psychiatric comorbidities of CM in population-based samples, finding increased levels of depression and anxiety disorders even in comparison with episodic migraine patients [11, 20]. One limitation of these studies was the use of self-report questionnaires rather than clinical interview in ascertaining psychiatric diagnosis. In the present study, we tried to overcome this using a validated structured clinical interview.

We found that up to a third of our patients in each setting had depression. A similar rate was described in the American Migraine Prevalence and Prevention (AMPP) study, a population-based survey based on mailed questionnaires [11]. In that study, depression was assessed by self-report of a physician diagnosis and by the Patient Health Questionnaire (PHQ-9)—depression module. One interesting result from the AMPP study was that CM patients were twice as likely to have depression as assessed by PHQ-9 in comparison with episodic migraine patients [30.2 and 17.2 % respectively; OR (95 % CI) = 2.0 (1.67 to 2.40), *p* < 0.001]. CM patients were also approximately twice as likely to report anxiety [30.2 vs. 18.8 % respectively; OR (95 % CI) = 1.8 (1.51 to 2.15), *p* < 0.001]. The International Burden of Migraine Study (IBMS) also found higher levels of anxiety and depression in CM when compared with episodic migraine [21].

Regarding anxiety syndromes, generalized anxiety disorder and phobias seem to be comorbid with the migraine spectrum [20–24]. In line with the literature, we found evidence for this association. Interestingly the frequency of obsessive–compulsive disorder was significantly high (between 20 and 25 %) in CM patients in comparison with its prevalence in the general population (2 %). Only few previous studies have pointed out this association between obsessive–compulsive disorder and migraine that may be associated with underlying serotonin system dysfunction [25].

Bipolar disorder is also comorbid with migraine; migraineurs without aura are 2.4 times more likely to have bipolar disorder type 1, and the ratio increases to 7.3 when the diagnosis is migraine with aura [13, 22]. For bipolar disorder type 2, values are 2.5 and 5.2, respectively [23]. We failed to detect this association, likely because of small size of the samples. Drug abuse has not been traditionally associated with migraine [22, 24], but a recent study reported that illicit drug abuse may be more frequent in migraine patients with depression or post-traumatic stress disorder [26]. We did not find evidence of this association in the current study.

Demographic profiles were similar in both groups, and the vast majority of individuals with CM were women. This over-representation was expected due to the epidemiological profile of migraine. It must be highlighted that the assessed community was from a small city, while the patients at the headache center mainly came from a large urban center. Nonetheless, studies of migraine that have enrolled subjects with different demographic features also found striking similarities regarding the risk of psychiatric comorbidities, once more pointing to shared biological factors as a plausible mechanism for the comorbidity [22, 24]. Specific genotypes coding D2 dopaminergic receptors, dysfunction in tyramine conjugation, changes in the metabolism of serotonin and catecholamines and in estrogen levels have been considered to explain the comorbidities [27–30].

Our study has several limitations and the major shortcoming is the small sample size. Difference in rates of some psychiatric disorders could be significant with a larger sample size. Therefore this report must be regarded as preliminary. It is worth mentioning, however, that we have comprehensively and systematically assessed almost all patients with CM from an entire population of a small city. We did not assess the differential disability associated with headache and psychiatric disorders in the individuals. Finally, our findings were not adjusted for other confounding factors, such as parameters of headache severity (intensity, duration associated symptoms), obesity, sleep disorders, use of psychotropic medication and household income. We partly justify these latter limitations by arguing that the demands on patients and resources in conducting these missing assessments could jeopardize the community assessment, since most interviews were conducted in participant households.

In conclusion, the present study suggests that psychiatric comorbidity in CM is elevated in the community and clinical settings, tending to be more common in CM patients from a headache center. Our finding must be confirmed by independent studies involving greater samples.
